# Semi-Synthetic Ingenol Derivative from *Euphorbia tirucalli* Inhibits Protein Kinase C Isotypes and Promotes Autophagy and S-Phase Arrest on Glioma Cell Lines

**DOI:** 10.3390/molecules24234265

**Published:** 2019-11-22

**Authors:** Viviane Aline Oliveira Silva, Marcela Nunes Rosa, Aline Tansini, Olga Martinho, Amilcar Tanuri, Adriane Feijó Evangelista, Adriana Cruvinel Carloni, João Paulo Lima, Luiz Francisco Pianowski, Rui Manuel Reis

**Affiliations:** 1 Molecular Oncology Research Center, Barretos Cancer Hospital, Barretos, São Paulo 14784-400, Brazil; vivianeaos@gmail.com (V.A.O.S.); nr.marcela2@gmail.com (M.N.R.); aline.cpom@hcancerbarretos.com.br (A.T.); olgamartinho@med.uminho.pt (O.M.); adriane.feijo@gmail.com (A.F.E.); drybiomedic@gmail.com (A.C.C.); 2 Life and Health Sciences Research Institute (ICVS), School of Health Sciences, University of Minho, 4710-057 Braga, Portugal; 3 ICVS/3B’s - PT Government Associate Laboratory, 4806-909 Braga/Guimarães, Portugal; 4 Laboratory of Molecular Virology, Departaments of genetics, IB, Federal University of Rio de Janeiro, Rio de Janeiro 21941-902, Brazil; atanuri1@gmail.com; 5 Medical Oncology, Barretos Cancer Hospital, Barretos, São Paulo 14784-400, Brazil; jpsnlima@yahoo.com.br; 6 Medical Oncology Department, A C Camargo Cancer Center, São Paulo 01509-010, SP, Brazil; 7 Kyolab Pesquisas Farmacêuticas, Valinhos, São Paulo 13273-105, Brazil; pianowski@kyolab.com.br

**Keywords:** glioma, cytotoxic activity, semi-synthetic derivative, ingenol, *Euphorbia tirucalli*, protein kinase C, autophagy

## Abstract

The identification of signaling pathways that are involved in gliomagenesis is crucial for targeted therapy design. In this study we assessed the biological and therapeutic effect of ingenol-3-dodecanoate (IngC) on glioma. IngC exhibited dose-time-dependent cytotoxic effects on large panel of glioma cell lines (adult, pediatric cancer cells, and primary cultures), as well as, effectively reduced colonies formation. Nevertheless, it was not been able to attenuate cell migration, invasion, and promote apoptotic effects when administered alone. IngC exposure promoted S-phase arrest associated with p21CIP/WAF1 overexpression and regulated a broad range of signaling effectors related to survival and cell cycle regulation. Moreover, IngC led glioma cells to autophagy by LC3B-II accumulation and exhibited increased cytotoxic sensitivity when combined to a specific autophagic inhibitor, bafilomycin A1. In comparison with temozolomide, IngC showed a mean increase of 106-fold in efficacy, with no synergistic effect when they were both combined. When compared with a known compound of the same class, namely ingenol-3-angelate (I3A, Picato^®^), IngC showed a mean 9.46-fold higher efficacy. Furthermore, IngC acted as a potent inhibitor of protein kinase C (PKC) activity, an emerging therapeutic target in glioma cells, showing differential actions against various PKC isotypes. These findings identify IngC as a promising lead compound for the development of new cancer therapy and they may guide the search for additional PKC inhibitors.

## 1. Introduction

Malignant gliomas are the most common and lethal primary brain tumors in humans [1]. Glioblastoma (WHO grade IV) is the most aggressive and frequent type of glioma [2,3] with dismal prognosis, even when current multidisciplinary treatment is used, with a median survival that has changed little in the last decades [4]. Comprehensive genetic analyses of Glioblastoma (GBMs) have identified a few mutations and pathways as therapeutic targets that contemplate EGFR, PI3K/AKT/mTOR, and Ras/MEK/MAPKinase [5,6]. Less explored, protein kinase C (PKC) proteins have emerged as possible targets due to their hyperactivity or overexpression, concomitant with the decrease of cell proliferation and invasion verified in preclinical glioma models [6,7,8,9]. Moreover, some clinical response in patients with refractory high-grade malignant gliomas was reported with PKC-inhibiting drugs [7,10,11,12]. Nevertheless, despite all biological and clinical advances, it is imperative to identify novel treatment strategies to glioblastoma outcome [4].

Several bioactive products that were derived from plants have been reported to prevent tumorigenesis of different types of tumors [13]. Thus, in the search for new therapies, research with natural products and on their anti-neoplastic mechanisms has emerged as an alternative and successful field [14]. *Euphorbia* species (Euphorbiaceae) have been used in traditional medicine as antimicrobial, antiparasitic, anticancer and other diseases [15]. Several secondary compounds are present in *Euphorbia* species extract and they are responsible for its properties [16,17]. Our group has carried out a bioprospecting program that evaluated the cytotoxicity of *E. tirucalli* compounds in a large panel of human tumor cell lines. We previously showed the cytotoxic effect of euphol, the main constituent of *E. tirucalli* latex, and its antitumor potential in glioma cell lines [18,19].

In addition to euphol, the genus *Euphorbia* also has diterpenes as important bioactive constituents some already approved for pre-cancerous conditions [20,21,22,23]. One diterpene that was approved for human use for the treatment of actinic keratosis, ingenol-3-angelate (I3A) (Picato^®^), from *Euphorbia peplus* demonstrated great antineoplastic potential evaluated in clinical trials for the effective treatment of basal cell carcinoma and squamous cell carcinoma through the modulation of PKCs signaling [24,25,26,27,28]. Some studies have also revealed diterpenes as promising modulators of multidrug resistance (MDR) in tumor cells as well as showing *in vivo* anti-inflammatory activity [29].

Recently, our group reported the cytotoxic potential of three new esters of semi-synthetic ingenol from *E. tirucalli* [20,21]. Among the three derivatives, ingenol-3-dodecanoate (Ingenol C—IngC) effectively promoted cytotoxicity and exhibited antitumoral properties. Besides, IngC showed higher efficacy when compared to I3A and ingenol 3,20-dibenzoate (IDB) from *E. esula* L on esophageal cancer cell lines, two important ingenol diterpenes that can promote PKC activation and anticancer activity [20,27,30]. However, the mechanism underlying IngC-induced antineoplastic effect is not largely understood.

Therefore, in this study, we unravel the antitumor properties of IngC derivative from *E. tirucalli* against glioblastoma-derived cells to provide a comprehensive view of its potential antitumor mechanisms.

## 2. Results

### 2.1. IngC promotes Cytotoxic Activity on Glioma Cell Lines More Effectively than Temozolomide but Their Combination Is Not Synergistic

The analyses of antitumor properties of IngC on glioma cells were expanded from our previous study [20]. Thus, the cytotoxicity was assessed by MTS assay in 13 glioma cell lines from commercial (adult and pediatric), primary, and one normal immortalized astrocytic cell line (Table 1). We observed that IngC exhibited dose and time-dependent cytotoxic effects on human glioma cells (Figure S1a). There was a heterogeneous profile to IngC, with each cell line exhibiting a distinct treatment response. The mean IC_50_ values among commercial cells was 6.86 μM, but significantly varied between individual cell lines, with more than a 68-fold difference in the IC_50_ values (IC_50_ range: 0.19–13.09 μM) (Table 1). Primary tumor cell cultures that were derived from glioblastoma surgical biopsies (HCB2 and HCB149) exhibited a more resistant profile to IngC in comparison with commercial cell lines (mean 15.98 μM) (Table 1).

**Figure 1:**
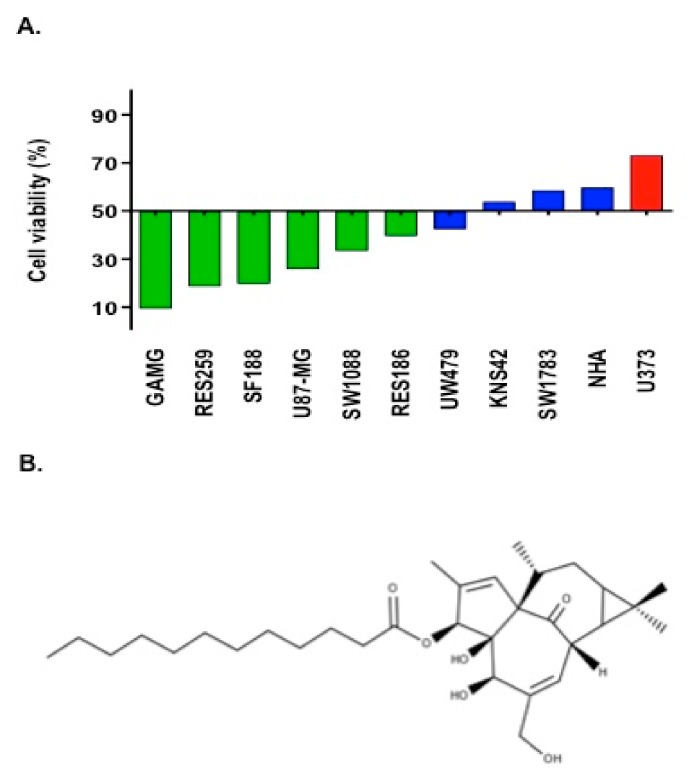
Chemical structures of modified ingenol derivative. (**A**) Cytotoxicity profile of 10 glioma cell lines and one normal human astrocyte exposed to IngC compound. Bars represent the cell viability at 10 μM of IngC. Colors represent the GI score classification: Green (HS = Highly Sensitive); Blue (MS = Moderate Sensitive) and Orange (R = Resistant). (**B**) ingenol-3-dodecanoate (IngC). http://www.chemspider.com/Chemical-Structure.28533061.html.

We adopted the criteria of growth inhibition (GI) at a fixed dose of 10 μM, which closely corresponds to the average IC_50_ value of all cell lines at initial screening, to better classify the response to IngC. At this fixed dose, we found that 9.1% (1/11) of cell lines were resistant, 36.4% (4/11) were moderately sensitive, and 54.5% (6/11) were classified as highly sensitive (Figure 1A and Table 1).

Furthermore, in comparison with temozolamide (TMZ), IngC showed a median of 106-fold increase in efficacy against glioma cell lines. Additionally, IngC demonstrated a higher selective cytoxicity index (SI) (0.37–39.05) than TMZ (0.11 to 1.13) (Table 1). However, the combination of IngC and TMZ exposure, promoted antagonistic effects (combination index >1) on 8/9 (88.89%) glioma cell lines (mean CI values: range: 1.13–1.9) (Table 1).

### 2.2. IngC Exhibts Higher Cytotoxic Activity than Other Ingenol-Ester Class on Glioma Cells

We further compared the antitumor activity of IngC with ingenol-3-angelate (I3A), the other compound of the same class adopted in clinical practice. For commercial cell lines, the mean IC_50_ values ranged from 0.19–13.09 μM for IngC, and from 0.01–95.15 μM for I3A, which indicated that IngC displayed a higher ingenol-ester cytotoxicity on glioma (Table 1).

### 2.3. Biological Properties of IngC in Cancer Cell Lines

#### 2.3.1. IngC Inhibits Proliferation and Induces S-Phase Arrest but Fails to Attenuate Migration and Invasion on Glioma Cells

**Figure 2:**
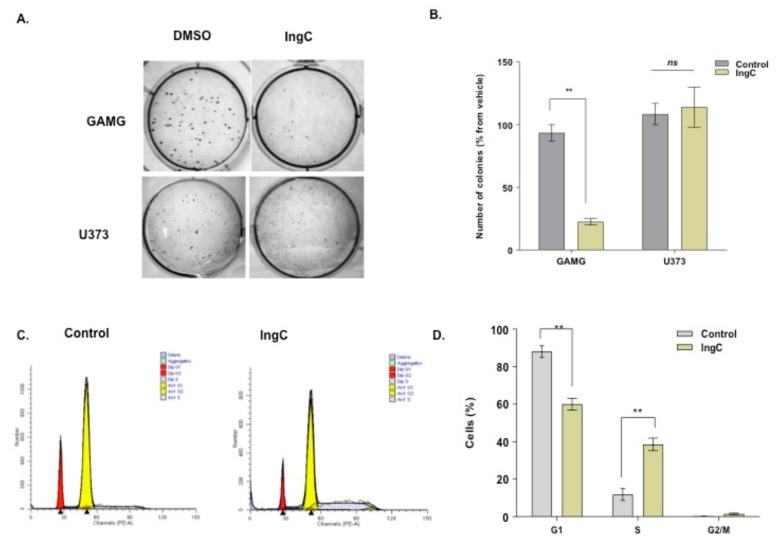
Effect of IngC in colony formation of glioma cells. (**A**) U373 and GAMG cells were seeded and grown in soft-agar medium containing the indicated compounds. (**B**) The number of colonies in each well was counted after 20 days of IngC treatment. The graphs are representative of at least two independent experiments performed in duplicate. *n.s*. means non-significant. (**C**) U373 cells (untreated and cells treated with IngC) were incubated for 72 h. Next, U373 cells were fixed with ethanol, stained with propidium iodide and cell cycle phase was analyzed by flow cytometry. (**D**) Results shown are the means ± S.D. of three independent experiments. *n.s.* means non-significant. ** *p* < 0.005.

Two representative cell lines for IngC sensitivity (GAMG line) and resistance response (U373 line) were selected to explore the biological role of IngC in cancer cells (Table 1). We first characterized the cell proliferation capabilities by colony formation assay and BrdU incorporation. IngC was able to reduce or inhibit significantly colony formation of GAMG cells, but not U373 (Figure 2A,B). Besides, IngC exhibited dose-dependent proliferation effects on the GAMG with an increase in BrdU- positive cells after 72 h (Figure S1b). IngC was less active amongst U373 cells, the greatest inhibition was observed at the highest IngC (30 μM) dose applied, being able to inhibit a little more than 30% of proliferation. These results suggest that IngC seems to have cytotoxic effects on the anchorage-dependent growth of both malignant glioma cell lines (Figure S1b).

The effect on the cell cycle profile was characterized by measuring the cellular DNA content. Flow cytometry revealed that IngC exerts significant effects on the cell cycle distribution. U373 cells that were treated with the compound were accumulated in S phase. U373 cells ranged from 11.3% in control to 38.3% when treated with IngC for 72 h (Figure 2C,D).

The impact of IngC on cellular migration and invasion was also evaluated, and no significant effect was observed in the IngC-sensitive or IngC resistant glioma cell lines at the time point and dose investigated (Figure S2a,b).

#### 2.3.2. IngC Induces Cell Death by Other Mechanisms, Not Apoptosis

**Figure 3:**
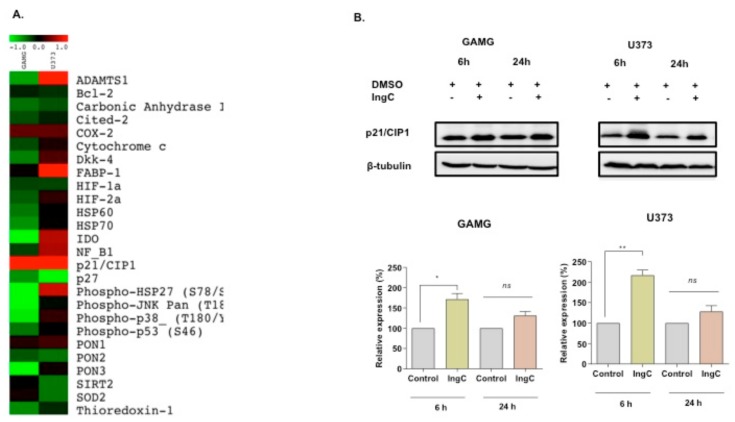
Effect of IngC on cell stress and cell cycle distribution on glioma cell lines. (**A**) A panel of 26 proteins related to cellular stress. Data represented by the heat maps show the proteins modulated after 6 h of IngC treatment (3X IC_50_ value) in glioma cells, GAMG and U373. The quantification and normalization of proteins was performed using the positive controls and untreated controls from the package *Protein Array Analyzer* of Image J software. (**B**) Cells were treated with IC_50_ concentrations of IngC (6 and 24 h) for the indicated time periods. GAMG and U373 cell lysates (20 μg per lane) were analyzed using immunoblotting with anti-p21/cip1. The tubulin was used as an internal control to normalize the amount of proteins applied in each lane. These data are representative of three independent experiments. *n.s.* means non-significant. * *p* < 0.05 and ** *p* < 0.005.

The effects of IngC on stress, apoptosis, and cell cycle were assessed by human apoptosis and cell stress proteome array. For IngC-sensitive cells (GAMG), the cell stress proteome array assay revealed that IngC exposure at 6 h resulted in a great downregulation of most stress-response proteins, including IDO, PON-3, p–HS27, p-JNKpan, and p-p38 (Figure 3A). In contrast, amongst the IngC-resistant U373 cell line, we observed, at 6h, a marked upregulation of ADAMTS-1, FABP-1, IDO, NF-κB1, and p–HS27 expression and minor changes in (upregulation) other proteins such as DKK4, HIF-2a, p-p38 and PON-1.

Remarkably, IngC also increased the levels of p21CIP1/WAF1 and, in a small proportion, increased COX2 expression in both cell lines (Figure 3A). The cell cycle modulation was also validated by immunobloting, which showed the up-regulated expression of cell cycle regulatory proteins p21CIP/WAF1 in both GAMG and U373 cells after 6 and 24 h (Figure 3B), corroborating the previous flow-cytometry analysis of IngC in cell cycle arrest.

**Figure 4:**
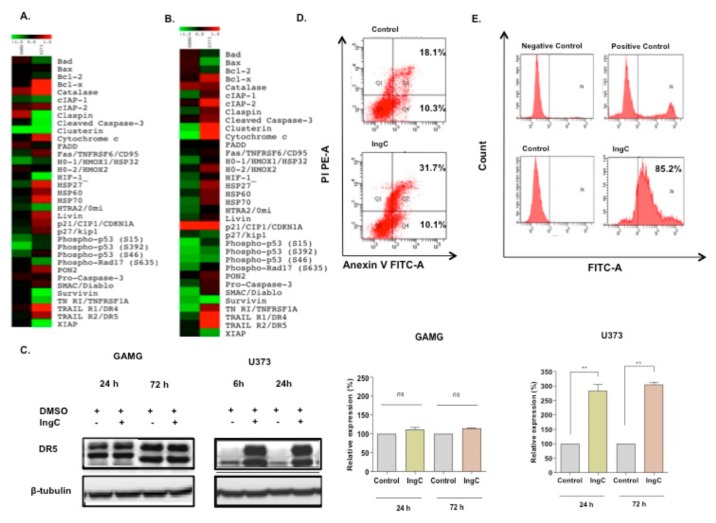
Effect of IngC on apoptosis pathway on glioma cell lines. (**A,B**) Panel of 35 proteins related to apoptosis. The data represented by the heat maps show the proteins modulated after 24 and 72 h of IngC treatment (3X IC_50_ value (13.09 μM) in glioma cells, GAMG and U373. (**C**) Cells were treated with 3X IC_50_ concentrations of IngC (24 and 72 h) for the indicated time periods. GAMG and U373 cell lysates (20 μg per lane) were analyzed using immunoblotting with anti-DR5. The tubulin was used as an internal control to normalize the amount of proteins applied in each lane. This data is representative of three independent experiments. (**D**) After the IngC treatment with 3X IC_50_ for 72 h, U373 cells were fixed, stained with annexin V-FITC/PI and analyzed by FACScan. Data shown are representative of three independent experiments. (**E**) After IngC treatment with 3X IC_50_ for 72 h, DNA fragmentation in U373 cell line was measured with the TUNEL assay using flow cytometry. The graphs are representative of at least three independent experiments performed in duplicate. *n.s.* means non-significant. ** *p* < 0.005

Moreover, using the human apoptosis proteome array (R&D systems), containing 35 different proteins related to apoptosis, we assessed the effect of IngC effect after 24 and 72 h on glioma cells (Figure 4A,B). At 24 h mark analysis, the (IngC-sensitive) GAMG cells had an upregulation of catalase, claspin and minor changes in cIAP-2 and FADD. Besides, Clusterin, p-P53 (S392) and at lower levels cIAP-1, H0-1/HM0X1/HSP32, pP53 (S15), and pP53 (S46) were markedly downregulated. At 72 h, IngC treatment promoted the expressive upregulation of p21CIP1/WAF1 and downregulation of cytocrome, p-P53 (S15), SMAC/DIABLO, survivin and TNFRSF1A in GAMG cells. Furthermore, IngC maintained the modulation of catalase, clusterin, pP53 (S392) and pP53 (S46). On the other hand, a greater modulation was observed in the IngC-resistant U373 cells at both times points. IngC downregulated the expression of several proteins at 24 h, especially: claspin, cleaved-caspase-3, clusterin, survivin, HIF-1, TNFRSF1A, and XIAP. In addition, proteins, such as Bad, BCL-2, cIAP-1, HTRA-2, and p-RAD17, were less intensely downregulated. The treatment also upregulated BcL-x, catalase, cytochrome C, HSP27, HSP60, HSP70, livin, P21, P27, PON2, TRAILR1/DR4, and TRAILR1/DR5. At 72 h, similarly to GAMG cells, p21CIP1/WAF1 was markedly upregulated in U373 cells. Furthermore, IngC kept the modulation of several stress proteins, such as BcL-x, catalase, cytochrome C, HSP27, HSP60, HSP70, cIAP-2, TRAILR1/DR4, and TRAILR1/DR5, and upregulated proteins before decrease as cIPA-1, claspin, cleaved-caspase-3, clusterin, and TNFRSF1A. These results were validated by immunobloting, which confirmed up-regulated expression of DR5 proteins in U373 cells after 24 and 72 h (Figure 4C).

The ability of IngC to induce cell death by apoptosis was also analyzed in glioma cells (sensitive or resistant ones) through flow cytometry (Figure 4D). As shown with proteomic profile arrays, no early apoptotic induction was revealed. The percentage of positive annexin V cells, indicative of early apoptosis, was not different from untreated control (10.3% to 10.1%, respectively), while the double positive annexin V/PI cells that are indicative of late apoptosis/necrosis revealed high percentual for IngC when compared to the untreated control (31.7% to 18.1%, respectively; Figure 4D). On the other hand, IngC treatment was able to induce DNA damage in these same lines, as evidenced by the Tunel assay. Close to 85.2% of the U373 cells were Tunel-positive after 72 h of IngC exposure, which suggests DNA fragmentation and a different type of cell death (Figure 4E).

#### 2.3.3. IngC Induces Autophagy in Glioma Cells

**Figure 5:**
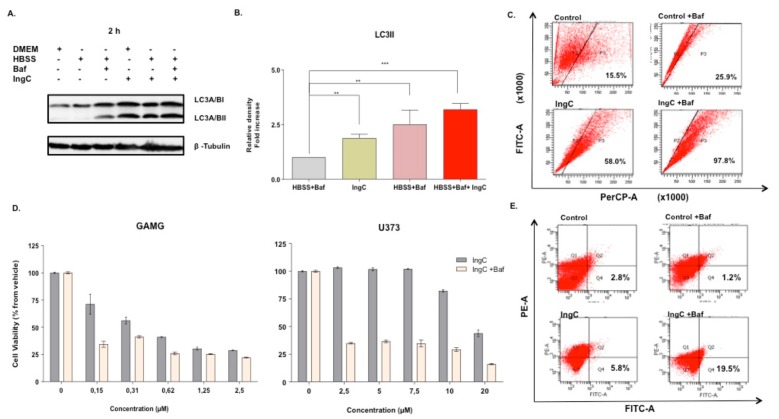
IngC promotes autophagy on glioma cells. (**A**) Cells were treated with the IC_50_ value of IngC for the indicated time periods. GAMG cell lysates (20 μg per lane) were analyzed using immunoblotting with anti-LC3. (**A**,**B**) are representative of three independent experiments with GAMG. Tubulin was used as an internal control to normalize the amount of proteins applied in the treatment without bafilomycin A1 (Baf). (**C**) Development of AVO in IngC-treated cells by detecting green and red fluorescence in acridine orange-stained cells using FACS analysis. U373 cells were treated with IngC (IC_50_ value), and Baf (20 nM) for 72 h. The graphs are representative of at least two independent experiments. FITC indicates green color intensity, while PerCP shows red color intensity. (**D**) Effect of baf on GAMG and U373 cell viability of IngC-treated cells. At 3 h after exposure to IngC, baf was added and cultured until 72 h and evaluated by MTS assay. The viability of the untreated cells was considered as 100%. Results shown are the means ± S.D. of three independent experiments. (**E**) Effect of Baf on IngC-induced apoptosis. After, IngC and bafilomicyn treatment for 72 h, GAMG cells were stained with annexin V-FITC/PI and analyzed by FACScan. Data shown are representative of three independent experiments. ** *p* < 0.005 and *** *p* < 0.0001

Given the importance of autophagy in cell death of gliomas, we wondered whether IngC could interfere in this process. For this, we evaluated autophagy-associated protein LC3-II expression. The GAMG and U373 cells were exposed to IngC for 2 h (IngC at IC_50_ value) and assessed by western blotting. GAMG cells exhibited a marked increase of LC3-II when compared to the untreated control cells after either IngC alone or when combined with Bafilomycin A1 (Baf) (Figure 5a,b). LC3-II levels expression in IngC alone and IngC combined to Baf were especially evident (2 and 2.9–fold increase, respectively) following 2 h of treatment. The presence of acidic vesicular organelles (AVOs), which are a non-specific marker for autophagy, was also analyzed. FACS scanning indicated an increase in the acridine orange positive cells when the cells were treated either with IngC alone or when combined with Baf (GAMG 10 nM). IngC-Baf combination led to greater formation of AVOs in U373 cells (58.0% in IngC versus 15.5% in controls and 97.8% in IngC-Baf versus 25.9% controls), thus indicating the development of AVOs suggestive of the autophagy process (Figure 5C).

U373 and GAMG were treated with different IngC concentrations for 72 h with the presence of Baf to investigate whether the inhibition of autophagy in late stages could affect the cytotoxicity of IngC. The viability of resistent U373 cells treated with IngC-Baf combination decreased in all concentrations tested as compared to the presence of Baf (20 nM) alone (Figure 5D). Next, we evaluated the double labeling of annexin V/PI in U373 cells to determine the effect of IngC combined with Baf in the apoptosis process. There was no appreciable change of positive cells to annexin V, indicative of early apoptosis, when comparing the control with IngC alone (2.8 to 5.8%) (Figure 5E). On the other hand, when compared to the Baf control, the treatment combining IngC to Baf increased the count of annexin V positive cells (1.2 to 19.5%), which suggesting that the cytotoxicity sensitivity was increased by the apoptosis mechanism (Figure 5E).

#### 2.3.4. IngC Exposure Inhibits Protein Kinase C Isotypes on Glioma Cells

**Figure 6:**
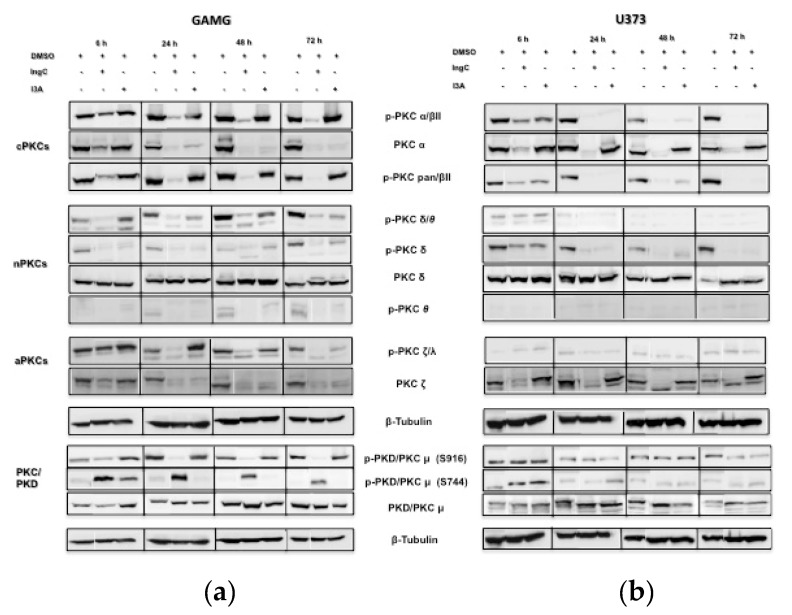
Effect of IngC on PKC isoforms in glioma cells. (**a**) GAMG and (**b**) U373 cells were incubated with the IC_50_ value for IngC, at 6, 24, 48 and 72 h. Controls were treated with DMSO alone (1%). Whole cell extracts from the same preparation were subjected to western-blotting analysis of PKC isoforms expressions. β-tubulin is shown as an internal control. Results shown are the means ± S.D of two independent experiments.

Multiple antitumor effects of diterpenes have been related to the direct modulation of PKCs, important proteins that are involved in cellular signal pathways [31]. We evaluated the PKC isotypes (conventional PKCs (cPKCs) to investigate the possible role of IngC in PKC signaling pathway: PKCα, p-PKCα/βII, p-PKCpanβII, novel PKCs (nPKCs): PKCδ, p-PKCδ, p-PKCδ/*θ, p-*PKC*θ*, and atypical PKCs (aPKCs): p-PKCζ/λ, PKCζ as well as PKD1/PKCμ, p-PKC PKDμ (Ser^916^), and p-PKC PKDμ (Ser^744^)) activation profile using immunoblotting (Figure 6). We also compared the IngC involvement in PKC activity with the known modulator I3A. IngC markedly downregulated the phosphorylation of most PKC isotypes (PKCalpha/beta, PKCpan/betaII, and PKC/PKDμ (Ser^916^)) as compared to I3A in GAMG cell line (Figure 6a). Moreover, similarly to IngC, I3A also decrease PKCδ/*θ*, PKCδ activation levels and the total expression of PKCα over time. Although less intensely, I3A also decreased the phosphorylation of PKCζ/λ and total levels of PKCζ over time (Figure 6a). In contrast to the general downregulation of PKC isoforms, the PKC/PKDμ (Ser^744^) isotype had transient phosphorilation by either IngC or I3A. PKC/PKDμ (Ser^744^) phosphorylation peaked at 6h with IngC and decreased over time, whereas I3A led to a short-lived peak, weaning off before 24 h (Figure 6a).

Further, in the U373 cell line, the PKC activity was modulated in the same way for IngC and I3A treatment, with exception of the total expression of PKCs α and ζ. Unlike GAMG cells, no modulation was found in the PKC/PKDμ (Ser^744^) isotype in the U373 cell line for both treatments (Figure 6b). These results show that IngC regulates PKCs activity with different responses according to cell sensitiveness.

## 3. Discussion

*Euphorbia tirucalli* is widely used as an anticancer drug in Brazilian folk medicine [15]. Our group recently identified *E. tirucalli* derived natural and semi-synthetic compounds, and showed their cytotoxicity effect in a wide-range of human cancer cells [19,20]. Particularly, a diterpene derived, ingenol-3-dodecanoate (IngC), exhibited the highest efficacy in several tumor cell lines, including gliomas [20]. In the present pre-clinical study, we extended and have gained a *comprehensive biological* insight into the underlying molecular mechanisms of IngC in gliomas. We showed that IngC is a major modulator of protein kinase C isotypes and it promotes autophagy and S-phase arrest in gliomas.

The antitumor potential of IngC was studied in a panel of 13 glioma cell lines, including commercial (adult and pediatric), primary cultures, and one normal human astrocyte cell line. IngC exhibited dose-time-dependent cytotoxicity on all glioma cell lines. The different models of glioma cell lines exhibited a heterogeneous profile of response to IngC. At a fixed dose of 10 μM, 9.1% (1/11) of cell lines were resistant, 36.4% (4/11) were moderately sensitive, while 54.5% (6/11) were classified as highly sensitive. This variation in response to IngC seems to be due to the innate differences in the molecular biology underlying adult, pediatric, and especially primary glioma cultures that could better mimic genomic heterogeneity from patients [19,32]. In our study, IngC showed more than twice the cytotoxicity to some cancer cell lines, mainly for GAMG (39.05) and SF188 (2.2) cells, when compared to normal cell lines, an interesting selectivity index preconized [33]. It was not possible to calculate the selectivity index for most of the cancer cell lines that were treated with TMZ, since this standard chemotherapeutic agent was more cytotoxic to the normal astrocyte cell line than to cancer cells.

Our results are in agreement with previous studies, which also demonstrated diterpenes cytotoxicity at micromolar range, such as ingenol 3,20-dibenzoate (IDB) from *E. esula* L in jurkat and breast cancer cells, and ingenol-3-angelate from *E. peplus* (I3A), in human melanoma, cervical cancer, and prostate xenografts [27,30,34,35]. Importantly, we showed that IngC presented higher efficacy when compared to I3A on glioma cells, suggesting this compound as promising against gliomas.

Studies addressing the cytotoxic effect of terpenes/diterpenes in glioma context are scarce. Kaurenoic acid, a bioactive diterpenoid that is present in *Mikania hirsutissima,* was evaluated in U87MG cells [36]. The concentrations used were much higher than the ones used in our study (30 to 70 µM for 24 to 72 h), despite previous reports showing the absence of cytotoxic effects on fibroblasts [37]. We also highlight that some semi-synthetic diterpenes are among the most cytotoxic drugs investigated, with an IC_50_ in the sub-nanomolar range [34].

Herein, we evaluated the intracellular signal pathways that were modulated by short-term exposure to IngC in glioma cells. Interestingly, stress and apoptosis panels of proteome arrays were the most modulated in U373, a drug-resistant cell line. We found that IngC promoted modulation in proteins related to stress and cell cycle, as well as anti-apoptotic and pro-apoptotic protein expression, with special reduction in the levels of pro-apoptotic BAX, BAD, TNRI/TNFRSF1A, and FASTNFR6/CD95, were observed for most of glioma cell lines overtime. In addition, anti-apoptotic proteins, including HSP27, HSP60, HSP70, livin, and PON-2, were upregulated in U373 cells. These results are consistent with flow cytometry studies, showing that changes in p21/CIPI WAf1 and anti-apoptotic factors were more pronounced, indicating that, in the conditions and cells tested, cell cycle arrest, but not apoptosis, contributed to the antiproliferative and cytotoxic effects of IngC in malignant glioma cell lines. Among the diterpenes described, IDB has relevant cell growth inhibition and apoptotic cell induction in jurkat cells and breast cancer cells [30,34]. Additionally, Lizarte and coworkers observed that Kaurenoic acid influences the regulation of several genes that are involved in the apoptotic pathway, including *c-FLIP, caspase 3, caspase 8*, and *miR-21* in U87 cells [26,27,28,35]. On the other hand, I3A promotes primary necrosis in melanoma, cervical cancer, and prostate xenografts, as well as inducing in vitro and in vivo models of colon cancer, apoptosis senescence, anti-inflammatory, and antitumor immunomodulatory properties in colon cancer. Although our data are discordant from these studies, these findings underscore our limited knowledge regarding the ingenoid pharmacophore and confirm that this paradoxical comportment is still poorly known [34,38].

We assessed the interplay of autophagy and IngC exposure due to the dual role of autophagy in cancer [39]. Temozolomide, the backbone of systemic therapy for glioblastoma, is reported to induce cell death by autophagic mechanisms [40]. Our study suggested that autophagy could play an important role in the antiproliferative mechanism of IngC. IngC induced LC3-II increase and marked formation of AVOs, indicating that this compound might activate an autophagic process. Moreover, the combined treatment of Baf and IngC potentiates the antitumor effect of IngC against malignant glioma cells by the autophagic vacuoles accumulation and apoptosis induction. Furthermore, the cell death that is induced by substances that suppress the autophagy pathway improved the effectiveness of TMZ and natural compounds in glioma cells [41]. These results are particularly important for GBM, since this tumor has been shown to be more resistant to cell death [42,43].

Protein kinase Cs (PKCs) contemplates a family of 14 known isozymes of serine/threonine-specific protein kinases, which are classified into three groups according to their interactions with calcium and diacylglycerol as cofactors; classical PKC (cPKC: α, β_1_, β_2_, and γ), novel PKC (nPKC: δ, ε, η, and θ), and atypical PKC (aPKC: ζ ι, ζII, ξ, v) [6]. PKD1 was initially recognized as a member of the protein kinase C (PKC) family and named PKCμ, however due to some particularities, it was reclassified. PKD1 is now a member of the protein kinase D (PKD) Family [44,45]. The role of PKC and PKD1 in processes that are relevant to neoplastic transformation, proliferation, apoptosis, and tumor cell invasion provides a potential suitable target for anticancer therapy [34,44,46], including glioma [6,9,10,11,12]. Provided that most of the biological effects of ingenol esters and derivatives are attributed to protein kinase C (PKC), such as co-carcinogenic and antitumor activity [31,34], we can assume a special interest in this field.

We compared IngC effects with I3A to gain more insight in this issue and found a marked potential inhibitory in the phosphorylation of most of PKC isotypes in sensitive GAMG cells by IngC, and a minor effect for I3A. In the U373 cell line, the PKC activity was modulated in the same way for IngC and I3A treatments, with the exception of total expression of PKCα and ζ. Moreover, IngC treatment in GAMG cells promoted the phosphorylation of PKC/PKDμ (Ser^744^) isotypes. A substantial PKC/PKDμ (Ser^744^) activation was also seen with I3A, but rapidly decreased. Of note, the results of I3A found in glioma are surprising, since I3A is a broad range activator of the classical (α, β, γ) and novel (δ, ε, η, θ) protein kinase C isoenzymes inducing direct pro-apoptotic effects in several malignant cells, including melanoma cell lines and primary human acute myelogenous leukemia cells [35,47]. In colon cancer, I3A induced the activation of PKCδ and reduced expression of PKCα, resulting in apoptosis [35]. These divergent biological responses are not completely understood, although it has long been recognized that there is marked heterogeneity in the patterns of biological behaviour induced by these agents’ analogs of diacylglycerol (DAG) [48]. The nature and position of the esters structure in the diterpenes ring could explain why analogs of DAG with different side chains induce different biological responses [34,48,49].

PKC isoforms differ, not only in their structure and substrate specificity and mode of activation, but also in their tissue distribution, subcellular localization, and biological functions [47,48]. The activation of PKC isoenzymes results in changes in their subcellular location following translocation. These observations clearly illustrate that the crosstalk between pro- and anti-apoptotic PKC isoforms is important, and the final effect of the PKC-agonist ingenol esters may therefore depend upon the balance between the various isoenzymes that are present within a tumor. In this sense, the drug might be less effective in those tumors with increased levels of the anti-apoptotic isoforms [48]. This issue could also explain why, although IngC treatment had inhibited PKC isoform activities that are involved in migration and invasion in glioma cells, such as PKCα and PKC/PKDμ, it was not able to inhibit cell invasion or migration in either glioma cell tested. These results corroborates with Do Carmo and coworkers, which revealed that, despite that PKCs have clear roles in GBM, the contribution of each isoform depends on residues phosphorylation, oncogenic mutations, cell environment, and type of stimuli/stress [6]. We emphasize that, although the literature and our findings provide the rationale for attempts to exploit PKC as a target for novel forms of treatment of GBM, our incomplete understanding of the cell- and tissue-specificity of the different PKC isoforms may lead to unexpected and/or undesired results in clinical practice becoming important subjects for further studies.

Finally, the combination of different agents with different targets of action might contribute to circumvent the chemoresistance of glioma cells. Recent studies had reported increased cytotoxicity that is induced by combinatory systems while using PKC inhibitors and TMZ [50]. In our study, combinatory therapy with IngC and TMZ promoted an antagonism effect in most of cell lines evaluated, which suggested that, although IngC promotes the inhibition of PKC activities, its administration with standard chemotherapy does not potentiate the effect of each other and could be related to their interaction with different isotypes of PKCs [47]. Further studies could focus on addressing these PKCs mechanisms in combination with other treatment modalities such as radiation and/or chemotherapy, which might help to refine our understanding of the glioma biology, enabling us to develop new therapeutic opportunities against this disease.

## 4. Materials and Methods

### 4.1. Cell Culture

Thirteen immortalized glioma cell lines (five adult and five pediatric glioma cell lines, two glioma primary cultures, and one normal human astrocyte) were obtained and cultivated, as indicated in Table 1. The two primary glioma cell lines were derived from surgical glioblastoma biopsies that were obtained at the Neurosurgery Department of the Barretos Cancer Hospital (São Paulo, Brazil). The local ethics committee approved the study protocol and patients signed an informed consent form [51]. The isolated cells were grown in DMEM medium under the same conditions described in Table 1. Besides mycoplasma analysis, all cell lines were authenticated by the Diagnostics Laboratory at the Barretos Cancer Hospital (São Paulo, Brazil) by short tandem repeat (STR) DNA typing, according to the International Reference Standard for Authentication of Human Cell Lines, as previously described [52]. Moreover, the established primary cultures were identified and confirmed by blood that was derived from the same patient.

### 4.2. Semi-Synthetic Ingenol

The synthesis of semi-synthetic ingenol-3-dodecanoate (IngC) from the sap of *E. tirucalli* was performed by Kyolab Laboratory (Campinas, Brazil) and provided by Amazônia Fitomedicamentos, Brazil (patent). The natural ingenol was altered by the addition of specific ester chains at carbon 3 of the core ring, as previously reported (Figure 1A) [20,21]. The ingenol synthetic derivative was diluted in dimethyl sulfoxide (DMSO) at 10 mM stock. The work dilutions were prepared to obtain a concentration of 1% DMSO. All of the dilutions were stored at −20 °C.

### 4.3. Cell Viability Analysis and IC_50_ Determination

The cytotoxic effect of IngC, its ingenol-ester analogue, (ingenol-3-angelate (I3A) (Adipogen (Switzerland)) and temozolomide (TMZ) (Sigma - **T2577**), *was* evaluated while *using* MTS assay (Cell Titer 96 Aqueous cell proliferation assay, Promega, Madison, WI, USA), following the manufacturer’s instructions. Cells were treated with increasing concentrations of IngC diluted in DMEM (0.5% fetal bovine serum (FBS)) and incubated for 72 h. Absorbance was measured in the automatic microplate reader Varioskan (Thermo) at 490 nm. The half maximal inhibitory concentration (IC_50_) was obtained by nonlinear regression while using GraphPad PRISM version 5 (GraphPad Software, La Jolla California USA), as previously described [18,19]. The growth inhibition (GI) was also calculated as a percentage of untreated controls, and its values were determined at a fixed dose of 10 μM (concentration closer to the average IC_50_ value of all cell lines at screening) [53]. Samples exhibiting more than 60% growth inhibition in the presence of 10 μM IngC were classified as highly sensitive (HS), as moderately sensitive (MS) when they were between 40 and 60%, and as resistant (R) when the values were lower than 40% of inhibition, as previous reported [53]. The selectivity index (SI) was obtained by dividing the IC_50_ value of a normal cell line (NHA) by a tumor cell line according to the National Cancer Institute (NCI) [19]. Significant SI values are considered to be greater than or equal to 2.0. [33]. The assays were performed in triplicate and repeated at least three times for each cell line.

### 4.4. Colony Formation-Assay

Anchorage-independent growth was performed while using a soft-type-agar assay, as reported [18]. Medium was changed every 72 h, and DMEM + 0.5% FBS containing IngC at IC_50_ concentrations values was added on GAMG, and U373 cell lines. The colonies formed were stained with 0.05% crystal violet for 15 min. and photo-documented. The analyses were performed by Image J Software. The assay was performed in two biological replicates and the experiments were done in duplicate.

### 4.5. Migration and Invasion Assays

Cell migration and invasion effects on GAMG and U373 cell lines were evaluated by wound healing assay and BD Biocoat Matrigel Invasion Chambers (354480, BD Biosciences), as previously described [54]. The shown images are representative of three independent experiments performed in triplicates. 

### 4.6. Cell Cycle and Cell Death Assays

Cell cycle and cell death assays were performed by flow cytometry, as previously described [19]. The cells were plated onto a six-well plate at a density of 1 × 10^6^ cells/well, allowed to adhere for at least 24 h and serum starved for 12 h. Additionally, the cells were exposed to 3X IC_50_ values of IngC for a period of 72 h in DMEM (0.5% FBS). The cell cycle distribution (G1, S, and G2/M) as well as double staining with Annexin V-FITC/PI and tunel assays, were determined with a flow cytometer *BD FACSCanto II* (*BD Biosciences*) and analyzed with the software BD FACSDiva (*BD Biosciences*), following the manufacturer’s recommended protocol. Approximately, 2 × 10^4^ cells were evaluated for each sample in both assays. The analyses were performed in experimental and biological triplicates.

### 4.7. Proteome Arrays

The relative protein expression levels of a panel of 35 proteins related to apoptosis and 26 proteins related to cellular stress were obtained while using the Proteome Profiler Human Apoptosis Array (R&Dsystems- #ARY009) and Proteome Profiler Human Cell Stress Array (R&Dsystems-#ARY018), according to the manufacturer’s instructions and as previously reported [54]. The selected cell lines (GAMG and U373) were treated with IngC for 6 and 24 h, for stress array and 24 and 72 h for apoptosis array while using a concentration equivalent to 3 x IC_50_ value of each glioma cell line. In addition, THE expression of some of the proteins was validated by western-blot analysis as described below.

### 4.8. Western Blotting

Protein expression after IngC treatment was evaluated by western blotting. Cells were plated onto a six-well plate at a density of 1 × 10^6^ cells/well, allowed to adhere at least 24 h, and then serum-starved in DMEM (0.5% FBS). The cells were exposed at IC_50_ values of IngC for several time points of 6, 24, 48, and 72 h in DMEM (0.5% FBS) and total protein was separated, as previously described for western blotting analysis [19]. Antibodies included anti-DR5, anti-p21/Cip1, anti-total PKCs (PKCα, PKCδ and PKCζ), anti-phosphorylated PKCs (p-PKC PKDμ (S916), p-PKC PKDμ (S744), p-PKCα/βII, p-PKCpanβII, p-PKCδ, p-PKCδ/*θ, p-*PKC*θ*, PKCζ/λ), and β-tubulin. All of the antibodies were diluted at 1:1000 and purchased from Cell Signaling Technology.

### 4.9. Autophagy Analysis: LC3 Expression, Acidic Vesicular Organelles (AVOs) and IngC and Autophagy Inhibitor Combination Effect on Glioma Cell Lines

GAMG and U373 cells were plated onto a six-well plate at a density of 5 × 10^5^ cells/ well, and allowed to adhere to evaluate the effect of IngC in the autophagy process. The growth medium was replaced by Hanks balanced salt solution (HBSS; Invitrogen) for cells starvation (two rinses in HBSS before being placed in HBSS). Cells were treated with 10 nM (GAMG) and 20 nM (U373) of bafilomycin A1 (Baf), to inhibit autophagy flux [19,41] or with IngC, while using a concentration equivalent to the IC_50_ of each cell line; or Baf and IngC combined; or DMEM alone as control. After 2, 6, or 24 h, the protein extracts was subjected to western blot analysis, as described above. For this, we used the primary polyclonal antibodies LC3A/B (dilution 1:1000; Cell signaling) and β-tubulin (dilution 1:5000; Cell Signaling Technology), as a loading control.

We also detected the acidic vesicular organelles (AVO) in the IngC-treated cells through vital staining with acridine orange, as reported previously [41]. The assay was performed according to the conditions for autophagy analysis, as mentioned above. Subsequently, 72 h after IngC exposure, cells were stained with acridine orange at a final concentration of 1 μg/mL for 15 min, washed twice in PBS 1X, and analyzed with a flow cytometer *BD FACSCanto II* (*BD Biosciences*) and BD FACSDiva software (*BD Biosciences*) following the manufacturer’s recommended protocol. These analyses were performed in experimental and biological triplicates.

GAMG and U373 cells were plated onto a 96-well plate at a density of 5 × 10^3^ cells/well, and allowed to adhere, and then, increasing concentrations of IngC were added to determine the effect of the autophagy inhibitor combine with IngC on cell viability. To inhibit autophagy, a fixed dose of Baf (10 nM for GAMG cells and 20 nM for U373 cells) was added to the culture 3 h after IngC treatment, as described [19]. The cell viability assay was evaluated after 72 h while using the Cell Titer 96 Aqueous test One Solution Cell Proliferation Assay (Promega), and measured as described above. The data were obtained and normalized relative to the average survival of untreated samples, or only treated with Baf. The analyses were performed in experimental and biological triplicates.

### 4.10. Drug Combination Studies

Combination studies from IngC and TMZ were performed with fixed concentrations (determined by the IC_50_ value) of the standard chemotherapeutic agent temozolomide, simultaneously exposed to increasing concentrations of IngC and evaluated by MTS assay as previously described above. Drug interactions were evaluated by the combination index while using CalcuSyn software version 2.0 (Biosoft; Ferguson, MO, USA), as previously described [55,56]. Synergy was defined as CI values that were significantly lower than 1.0; antagonism as CI values significantly higher than 1.0; and, additive as CI values that are equal to 1.0 [55,56] at drug IC_50_ value for each cell line.

### 4.11. Statistical Analysis

Data were expressed as the mean ± standard deviation (SD) of three independent experiments. We applied the Student’s t-test for comparing two different conditions, whereas two-way analysis of variance (ANOVA) was used for assessing the differences between more groups. *p*-values <0.05 were considered to be significant. All of the analyses were performed whle using the aforementioned GraphPad PRISM version 8 (GraphPad Software, La Jolla, CA, USA).

## 5. Conclusions

Our current findings add a new layer of complexity to understand the diterpene mechanism, including its modulation of the autophagic process and providing a comprehensive view of IngC in glioma. Importantly, this study supports ongoing efforts targeting PKC proteins in cancer therapy with IngC and its indicates as lead semi-synthetic diterpene based PKC inhibitors, which represents a novel and promising antitumor drug to target cancer cells.

## Figures and Tables

**Table 1:** Semi-synthetic ingenol derivative (IngC), ingenol-3 angelate (I3A) and temozolomide (TMZ) values of half maximal inhibitory concentration (IC_50_), drug combination studies in glioma cell lines, cell lines origin, and culture conditions.

Cell Line	IngC IC_50_ ±S.D (μM)	I3A IC_50_ ±S.D (μM)	TMZ IC_50_ ±S.D (μM)	Combination Index (CI) *** TMZ+IngC	IngC Growth Inhibition in % at 10 μM *	IngC Growth Inhibition (GI) Score *	S.D	IngC SI **	TMZ SI **	Origin	Culture Conditions	Tumor Type
**U87-MG**	4.02 ± 2.29	95.15 ± 14.35	746.76 ± 3.15	1.46	74.18	HS	10.46	1.85	0.15	ATCC	DMEM + 10% FBS + 1% P/S	**Adult glioma**
**U373**	13.09± 0.84	76.39 ± 19.24	544.75 ± 1.53	0.80	26.88	R	8.19	0.57	0.20	Kindly provided by Dr. Joseph Costello	DMEM + 10% FBS + 1% P/S	
**GAMG**	0.19 ± 0.05	0.010 ± 0.13	97.00 ± 2.05	*UD*	90.58	HS	1.32	39.05	1.13	DSMZ	DMEM + 10% FBS + 1% P/S	
**SW1088**	7.48 ± 0.47	87.62 ± 0.33	979.2 ± 4.00	1.20	66.64	HS	10.18	0.99	0.11	ATCC	DMEM + 10% FBS + 1% P/S	
**SW1783**	7.4 ± 0.93	91.12 ± 1.59	>1000	1.83	41.39	MS	0.63	1.00	*UD*	ATCC	DMEM + 10% FBS + 1% P/S	
**RES186**	10.76 ± 2.6	89.56 ± 3.62	714.75 ± 7.08	1.35	60.4	HS	23.2	0.69	0.15	kindly provided by Dr. Chris Jones	DMEM + 10% FBS + 1% P/S	**Pediatric glioma**
**RES259**	5.28 ± 1.54	78.15 ± 23.66	206.05 ± 6.09	1.13	81.4	HS	5.51	1.41	0.53	kindly provided by Dr. Chris Jones	DMEM + 10% FBS + 1% P/S	
**KNS42**	8.10 ± 1.17	84.84 ± 34.86	>1000	1.9	46.14	MS	5.07	0.92	*UD*	kindly provided by Dr. Chris Jones	DMEM + 10% FBS + 1% P/S	
**UW479**	8.89 ± 0.86	72.63 ± 12.45	>1000	1.2	57.57	MS	8.61	0.83	*UD*	kindly provided by Dr. Chris Jones	DMEM + 10% FBS + 1% P/S	
**SF188**	3.38 ± 1.24	0.039±0.02	>1000	1.80	80.24	HS	2.8	2.20	*UD*	kindly provided by Dr. Chris Jones	DMEM + 10% FBS + 1% P/S	
**HCB2**	11.79± 1.04	*ND*	*ND*	*ND*	59.4	MS	*ND*	0.63	*ND*	Barretos Cancer Hospital	DMEM + 10% FBS + 1% P/S	**Primary culture**
**HCB149**	20.16± 1.34	*ND*	*ND*	*ND*	20.3	R	*ND*	0.37	*ND*	Barretos Cancer Hospital	DMEM + 10% FBS + 1% P/S	
**NHA**	7.42 ± 2.46	37.59 ± 8.34	110.5 ± 1.05	*ND*	59.77	MS	*ND*			ECACC	DMEM + 10% FBS + 1% P/S	**Normal Human** **Astrocyte**

* **Growth inhibition** (GI) was calculated as a percentage of untreated controls, and its values were determined at a fixed dose of 10 μM. Samples exhibiting more than 60% growth inhibition in the presence of 10 μM IngC were classified as highly sensitive (HS); as resistant (R) when showing less than 40%; and as moderately sensitive (MS) when showing between 40 and 60% growing inhibition. ** The selectivity index (SI) is the ratio between the IC_50_ values for NHA (IngC IC_50_ = 7.42 ± 2.46 and TMZ IC_50_ = 110.5 ± 1.05 μM) and those for the glioma cell lines. *** The Combination Index (CI) was analyzed using CalcuSyn Software version 2.0. The CI value significantly lower than 1.0, indicates drug synergism; CI value significantly higher than 1.0, drug antagonism; and CI value equal to 1.0, additive effect. *UD* = undetermined; *ND* = not determined; * IngC (ingenol-3-dodecanoate); I3A (ingenol-3-angelate); TMZ (temozolomide); FBS (fetal bovine serum). ATCC (American Type Culture Collection); DSMZ (German Collection of Microorganisms and Cell Cultures; ECACC (European Collection of Authenticated Cultures).

## Supplementary Materials

The following are available online. Figure S1: Time and concentration cytotoxic effect of IngC exposure on human cancer cell lines. Figure S2: Effect of IngC on migration and invasion of glioma cells.

## Abbreviations

AVOs	Acidic Vesicular Organelles
ANOVA	Analysis of variance
ATCC	American Type Culture Collection
Baf	bafilomycin A1
CI	Combination index
DNA	Deoxyribonucleic acid
DSMZ	German Collection of Microorganisms and Cell Cultures
DMEM	Dulbecco’s modified Eagle’s medium
DMSO	Dimethyl sulfoxide
ECACC	European Collection of Authenticated Cultures
FBS	Fetal bovine serum
FDA	Food and Drug Administration
GBM	Glioblastoma
GI	growth inhibition
g	Gram
IC_50_,	Half maximal inhibitory concentration
IngC,	ingenol-3-dodecanoate; I3A, ingenol-3-angelate
IDB	ingenol 3,20-dibenzoate
mL	Milliliter
MTS	3-(4,5-dimethylthiazol-2-yl)-5-(3-carboxymethoxyphenyl)-2-(4-sulfophenyl)-2H91tetrazolium)
NCI	National Cancer Institute
PKC	Protein kinase C
P/S	Penicillin/streptomycin solution
RPMI-1640	Roswell Park Memorial Institute
SD	Standard deviation
STR	Short tandem repeat
TMZ	temozolamide
WHO	World Health Organization
uM	Micromolar
