# Supplementary material for: Semi-Synthetic Ingenol Derivative from Euphorbia tirucalli Inhibits Protein Kinase C Isotypes and Promotes Autophagy and S-Phase Arrest on Glioma Cell Lines
Source: Molecules. 2019 Nov 22;24(23):4265. doi: 10.3390/molecules24234265 (PMC6930609; doi:10.3390/molecules24234265)
Supplement: Supplementary file 1 [file molecules-24-04265-s001.pdf]

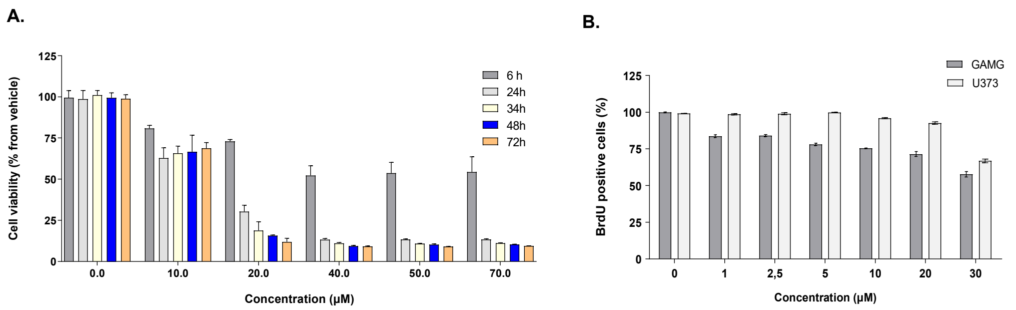

Figure S1. Time and concentration cytotoxic effect of IngC exposure on human cancer cell line. (**a**) Cell viability was measured with MTS assay after 72 h of IngC treatment. Cell viability of the untreated cells was regarded as 100%. Results shown are the means ± S.D of three independent experiments. (**b**) Effect of ingenol C on glioma cell proliferation. Cell proliferation was measured with BrdU assay after 72 h of IngC treatment. The proliferation of the untreated cells was regarded as 100%. Results shown are the means ± S.D of three independent experiments.

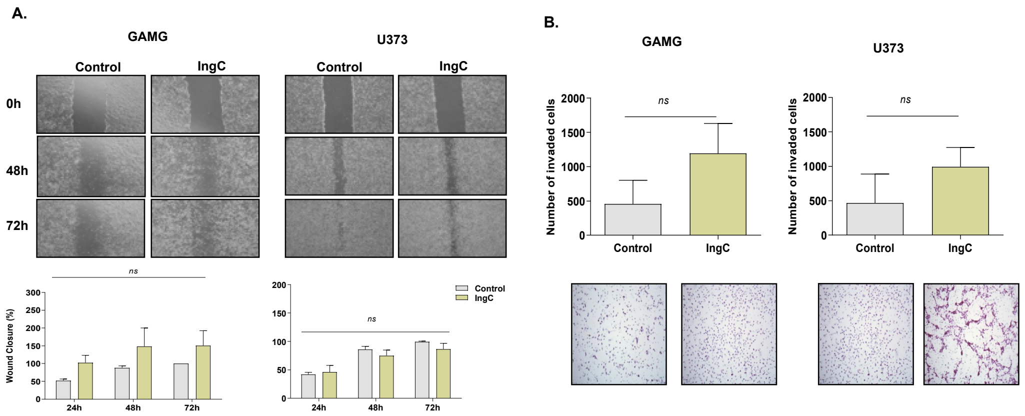

Figure S2. Effect of IngC on migration and invasion of glioma cells. (**a**) In the wound healing migration assay, a standardized scratch (wound) was applied to monolayers of GAMG and U373 cells and digital images were taken at several time points (0, 24, 48 and 72 h) in the same area. The distance in pixels was calculated and the percentage was calculated at time 0 h. The figures and graphs are representative of three independent experiments. (**b**) GAMG and U373 cells were seeded into serum-free medium containing the indicated compounds in a matrigel insert. The number of cells in each membrane was determined after 24 h of treatment. The graphs are representative of three independent experiments. *n.s.* means non-significant.
